# COVID-19 Reinfection in a Young Medical Doctor: A Case Report

**DOI:** 10.31729/jnma.6450

**Published:** 2021-07-31

**Authors:** Dhruba Gaire, Muneshwar Sah, Bishnu Singh

**Affiliations:** 1 Bir Hospital, National Academy of Medical Sciences, Kathmandu, Nepal; 2 Himal Hospital Pvt. Limited, Gyaneshwor, Kathmandu, Nepal

**Keywords:** *COVID-19*, *reinfection*, *new variants*, *reactivation*, *vaccination*

## Abstract

There is hardly any report of reinfection due to coronavirus disease 2019 (COVID-19) in medical professionals from Nepal. We report a case of a 32-year-old doctor with COVID-19 reinfection. Symptoms during the first infection were mild. After one month, he was reinfected and developed diarrhea as well as a continuous high fever. His d-dimer and ferritin were much increased. Computed tomography chest showed bilateral lymph nodes, minimal pleural effusion, and scattered linear fibrosis. After discharge, his depression and myalgia persisted for one month. During reinfection, his symptoms were more severe and cost of treatment was almost eight times his monthly salary and he could not work for six weeks. Possible reasons for severe reinfection and differential diagnoses like cytokine storm, multisystem inflammatory syndrome, reactivation of COVID-19, and infection due to new variants were discussed. Whether infected or vaccinated or not, all should take recommended vaccination and primary-preventive as well as health-promotive measures.

## INTRODUCTION

Many respiratory tract infections in human beings are often caused by four types of endemic corona viruses and reinfection with them occur eventually.^1^ Corona virus disease 2019 (COVID-19) is an entirely new type of corona virus and whether infection confers immunity to reinfection or how frequently reinfection will occur is uncertain.^1,2^ Since the pandemic began, almost 30% of total COVID-19 deaths in Nepal have occurred in people aged 15 to 55 years.^3^ There is hardly any report of re-infection due to COVID-19 in medical professionals from Nepal. We report here a re-infection case in a young doctor.

## CASE REPORT

After a week of COVID-ward duty, a 32 year old healthy medical doctor without any comorbidity developed mild fever with intermittent maximum temperature of 100° F associated with mild body ache. He did not have cough, shortness of breath, diarrhea, or taste or smell disturbance. His body mass index (BMI) was 26.67 kg/m^2^. Reverse transcription-polymerase chain reaction (RT-PCR) for COVID-19 done on oropharyngeal and nasopharyngeal swab on September 24, 2020 was positive (cycle threshold value 12.3, 17.4, and 18.1). He was admitted in hospital for observation and managed with vitamin supplementation and hydroxychloroquin 200 mg twice daily for five days. He was subsequently transferred to institutional quarantine for two weeks. Saturation was maintained throughout and there was no need of oxygen therapy. His repeat RT-PCR done on oropharyngeal and nasopharyngeal swab was negative on October 8, 2020. After discharge, he was alright except for mild fatigue. He resumed the regular duties after being negative for COVID-19.

After 25 days of having negative test of COVID-19, he again developed high grade fever with maximum temperature of 104° F associated with chills and rigor. He also had dry cough with no hemoptysis and no shortness of breath. He had diarrhea with passage of loose stool initially 2-3 episodes per day followed by more frequent stool after treatment with antibiotic. He had severe myalgia and weakness. Chest radiograph was normal. The presence of re-infection with COVID-19 was confirmed by RT-PCR (cycle threshold value 25.6, 27.5) done on November 2, 2020. His nasopharyngeal swab for influenza virus was negative. He was managed with oral electrolyte solution, azithromycin 500mg daily, paracetamol and vitamins. However the fever continued with no relief with medication and he also developed severe headache, and asthenia. He was admitted in a hospital and treated with injection paracetamol and ceftriaxone, but the fever didn't subside. His blood sugar, urea, creatinine, and electrolytes remained normal. His Chest radiograph was normal. He maintained oxygen saturation between 94% and 97% on room air. However, his white blood cell count started increasing and related tests of inflammatory reactions also increased much (Table 1).

**Table 1:** Investigations during reinfection with COVID-19.

Parameters	Results	Reference range
WBC count (neutrophil %)	17,860 (94%)	4000-11000/cumm
CRP	140.9mg/l	<6mg/l
LDH	574U/L	225-450U/L
APTT	45.7	25-39 sec
D-dimer	>10,000ng/ml	<500ng/ml
Ferritin	>2000ng/ml	Male 22.0 - 322ng/ml
Procalcitonin	5.02ng/ml	<0.5ng/ml

Rapid antibody detection test for COVID-19 was negative. His antibiotics were changed to injection meropenam, teicoplanin and doxycycline. Contrast enhanced computer tomography pulmonary angiography (CECT PA) done on November 6, 2020 showed normal CT pulmonary angiography, prominent lymph nodes in bilateral and subcarinal region, largest of which measures 10 mm, and bilateral minimal pleural effusion along with minimal linear fibrotic densities were noted in the right middle lobe, left lingual, superior, lateral basal and posterior basal segment of right lower lobe, superior, anteromedial basal and posterior basal segment of left lower lobe. There was no old CT chest available to compare. Injection Remdesivir was started, but it had to be stopped after three days due to transient AST and ALT elevations, to 203 and 227 respectively, and other deranged liver function tests with hypoproteinemia. His fever was continuous associated with chills and rigors for first 6 days which later became intermittent with relatively small spikes and by 10th day, he was afebrile. Diarrhea stopped at seventh day. His repeat nasopharyngeal swab for RT-PCR test done on November 8, 2020 was negative and he was discharged.

He had insomnia and was very fearful and anxious for almost two weeks following discharge from hospital. He used to have vivid recall of memories of his stay in intensive care unit with deaths due to COVID-19 occurring around. His myalgia persisted for almost one month. Values of various biochemical tests became normal within one month on routine follow-up. He was depressed for nearly one month with loss of interest in study and work avoiding any news of COVID-19 and people. He joined his duty after one month of discharge. The cost of treatment during the reinfection of COVID-19 was around rupees three hundred thousand, which was almost 8 times his monthly salary. During the reinfection and recovery from the illness, he could not work for one and half month. Almost five months after discharge, he still gets anxious when he hears the news of arrival of second wave of COVID-19 and his co-workers getting infected.

## DISCUSSION

It has been known that protective immunity is shortlived after seasonal corona viruses.^4,5^ The pandemic due to due to the novel corona virus now known as severe acute respiratory syndrome corona virus-2 (SARS-CoV-2) began since one and half year ago. The World Health Organization named the disease corona virus disease 2019 (COVID-19).^6^ Since the beginning of the COVID-19 pandemic, the issue of reinfection has been raised and discussed. We reported the case of a young medical doctor with COVID-19 reinfection in Nepal with more severe symptoms and higher cost of management. Thought most of the COVID-19 reinfections that have been reported have been milder than first encounters with the virus, reinfection can be severe and deaths have been reported.^1,4^

Like the first confirmed case of COVID-19 reinfection in the USA,^7^ our case also had worse disease than the first infection. There could be several reasons for more severe symptoms during reinfection. One risk factor of severe disease in our patient was high BMI of 26.67kg/m^2^ which was considered as obesity for Asian population.^8^ The other difference during infection and reinfection was the use of hydroxychloroquin during first infection only. There is no clear and well acceptable evidence of efficacy of hydroxychloroquine or chloroquine in the treatment of COVID-19.^9^ The data of frequency and severity of COVID-19 infection and disease in patients who were already receiving long term hydroxychloroquin or chloroquin for rheumatology and other indications may provide further evidence regarding their efficacy. Further it is speculated that those who experience the mildest symptoms in their initial infection may have a higher likelihood of reinfection, perhaps because they might not have developed adequate immune response the first time.^1^ This is consistent with the negative rapid antibody detection test for COVID-19 during the reinfection period in our patient who had milder symptoms during the first infection, though there are limitations of the rapid antibody detection test. Immunosuppressed patients may also not mount adequate immune response to the first infection and may be more susceptible to reinfection.^1^ Overall the occurrence of reinfection due to COVID-19 in the community remains a risk. However, even if reinfections are happening frequently in the community, the magnitude of reinfections may not be appreciated unless the studies are specifically designed to find out such problem.^1^

There are some differential diagnoses and conditions to consider in the case report. One differential diagnosis to consider is cytokine storm which a proportion of patients progress to at later stage of COVID-19.^10^ However our patient had negative RT-PCR test after the first illness due to COVID-19 and he had resumed normal activity for about four weeks when he had re-infection. The other related illness to consider is multisystem infammatory syndrome in adults (MIS-A). However MIS-A, like the multisystem infammatory syndrome in children (MIS-C), develops several weeks following confirmed or suspected COVID-19 infection and patients with MIS-A or MIS-C have negative RT-PCR test for COVID-19.^10^ In our patient, the RT-PCR test was positive during the reinfection period after becoming negative after the first infection with COVID-19. The third differential diagnosis to consider is reactivation of COVID-19 which is difficult to rule out. To certainly differentiate the reinfection from reactivation, the genomes of the viruses from both the samples collected during infection and reinfection would need to be shown to be different for it to be a reinfection.^1,11^ The large genomic structures of the COVID-19 could cause them to remain in the body at low enough levels to remain undetected which may be reactivated later.^1^ The fourth differential diagnosis is whether the reinfection is due to infection due to the new variants of COVID-19. Reinfection is likely due to the genetic drift in the COVID-19 such that the variant may elude the immune system in the body.^1^

There are implications of COVID-19 reinfection to consider by the health professionals and others. Reinfection can happen. Reinfection can severely affect the person. Even if the symptoms are mild, they can transmit the infection to their family members or colleagues who can develop severe disease due to primary infection or reinfection. COVID-19 vaccine generates more consistent adequate immune response than COVID-19 infections.^1,12^ Vaccination should, thus, be done as per the availability and guidelines whether one is infected or not. However, COVID-19 infection may occur even after vaccination.^13^ The personal preventive measures like physical distancing, hand hygiene, wearing mask, and using personal protective equipment need to be considered by all whether one was infected or nor, or vaccinated or not. Apart from the personal preventive measures and vaccination, various measures that promote physical and mental health and may prevent occurrence of COVID-19 infection or its progression to complications should also be considered.^14^ Such health promotive measures which may prevent the occurrence of COVID-19 infection or its comorbidity and complications may not get adequate attention of the people unless they are emphasized by the national and global health agencies.^14^ Thus to prevent infection or reinfection due to COVID-19, vaccination and various personal preventive and health promotive measures need to be considered by all whether they are already infected, reinfected or vaccinated or not. Health care professionals are at high risk of getting COVID-19 infection and reinfection. Due priority needs to be given by the hospital management and other stakeholders to vaccination and such personal preventive and health promotive measures to protect the health care professionals.
